# Role of HOXA9 in solid tumors: mechanistic insights and therapeutic potential

**DOI:** 10.1186/s12935-022-02767-9

**Published:** 2022-11-14

**Authors:** Ling Tang, Lin Peng, Chao Tan, Huai Liu, Pan Chen, Hui Wang

**Affiliations:** 1 Hunan Key Laboratory of Translational Radiation Oncology, Hunan Cancer Hospital and The Affiliated Cancer Hospital of Xiangya School of Medicine, Central South University, Changsha, 410013 Hunan China; 2 Department of Radiation Oncology, Hunan Cancer Hospital and The Affiliated Cancer Hospital of Xiangya School of Medicine, Central South University, Changsha, 410013 Hunan China; 3 Graduate Collaborative Training Base of Hunan Cancer Hospital, Hengyang Medical School, University of South China, Hengyang, 421001 Hunan China

**Keywords:** HOXA9, Tumor, Mechanism, Therapeutic potential

## Abstract

HOXA9 functioning as a transcription factor is one of the members of HOX gene family, which governs multiple cellular activities by facilitating cellular signal transduction. In addition to be a driver in AML which has been widely studied, the role of HOXA9 in solid tumor progression has also received increasing attention in recent years, where the aberrant expression of HOXA9 is closely associated with the prognosis of patient. This review details the signaling pathways, binding partners, post-transcriptional regulation of HOXA9, and possible inhibitors of HOXA9 in solid tumors, which provides a reference basis for further study on the role of HOXA9 in solid tumors.

## Background

Homeobox genes were firstly discovered when identifying the characterization of several functional genes in Drosophila development [1]. Members of the HOX family have a well-conserved DNA binding homeodomain which have indispensable roles in managing the expression of genes during early development [2]. At present, 39 HOX genes were identified and classified into four clusters including HOXA cluster, HOXB cluster, HOXC cluster, and HOXD cluster [3]. Some biological processes such as hematopoiesis [4], vascularization [5], and fertility [6] needs the participation of HOX gene family. Function as transcription factors, abnormal activation of HOX genes leads to the dysregulation of downstream signal pathways resulting in the development of various diseases [7]. Indeed, many HOX genes are considered as key regulators for their implications in gene translocation, gene mutation, or improper gene expression in tumor, which play a critical role in the proliferation, invasion, and metastasis stages of tumor [8].

HOXA9, a member of HOX family belonging to the HOXA cluster, is often studied in acute myeloid leukemia (AML), which is linked to proliferation, differentiation, and progenitor self-renewal maintenance [9]. HOXA9 is considered as the most correlative marker of poor prognosis and a driver in AML [10], indicating that HOXA9 is a potential target in AML. Additionally, research also focused on the role of HOXA9 in solid tumors such as ovarian cancer [11], and breast cancer [12]. At present, no systematic reviews described the role of HOXA9 in various solid tumors, signaling pathways associated with the regulation of HOXA9, binding partners, the post-transcription modification of HOXA9, and inhibitors of HOXA9.

Therefore, we primarily focus on understanding the roles of HOXA9 in solid tumors in this review mainly involving the signaling pathways, binding partners, post-transcription modification including microRNAs (miRNAs), non-coding RNAs (ncRNAs), and methylation of HOXA9. Finally, we summarize the possibility of inhibitors based on interfering HOXA9 expression.

## Structure and function of HOXA9

Located on the short arm of human chromosome 7 (7p15-p14), HOXA9 is composed of exon AB, exon CD, and exon II respectively. Exon AB contains a 358 bp coding region, and the last 150 bp completely overlaps with part of human fetal HOXA9 cDNA sequences. Exon CD has a coding region of 586 bp, and the last 90 bp is matched with the remaining part of cDNA sequences. Furthermore, the alternative splice sites of exon CD existing in humans and other species results in different transcripts which might play a crucial role in human disease [13]. Among these exons, exon II is a particularly important region due to the ability for coding the DNA binding domain, and together with the 3′ UTR makes up the most shared region. The promoter region of HOXA9 gene is rich in CpG structure, and the encoded protein has a typical helix-turn-helix structure that recognizes specific TTAGAC sequences [14], while the binding ability after recognition is not strong, and HOXA9 protein binding with transcription factors such as PBX through heterodimerization enhances the specificity of their binding [15]. Two different HOXA9 proteins encoded by HOXA9 share common exon II which encodes the DNA binding domain. Transcript HA-9A is only expressed during development, while the other canonical HOXA9 transcript (HA-9B) containing two exons is expressed in multiple tissues in fetal and adult organisms, especially in endothelial cells. HA-9B is consists of exon CD and the common exon II together with 3′ UTR, and its expression is well-conserved crossing of various species [16].

**Fig. 1:**
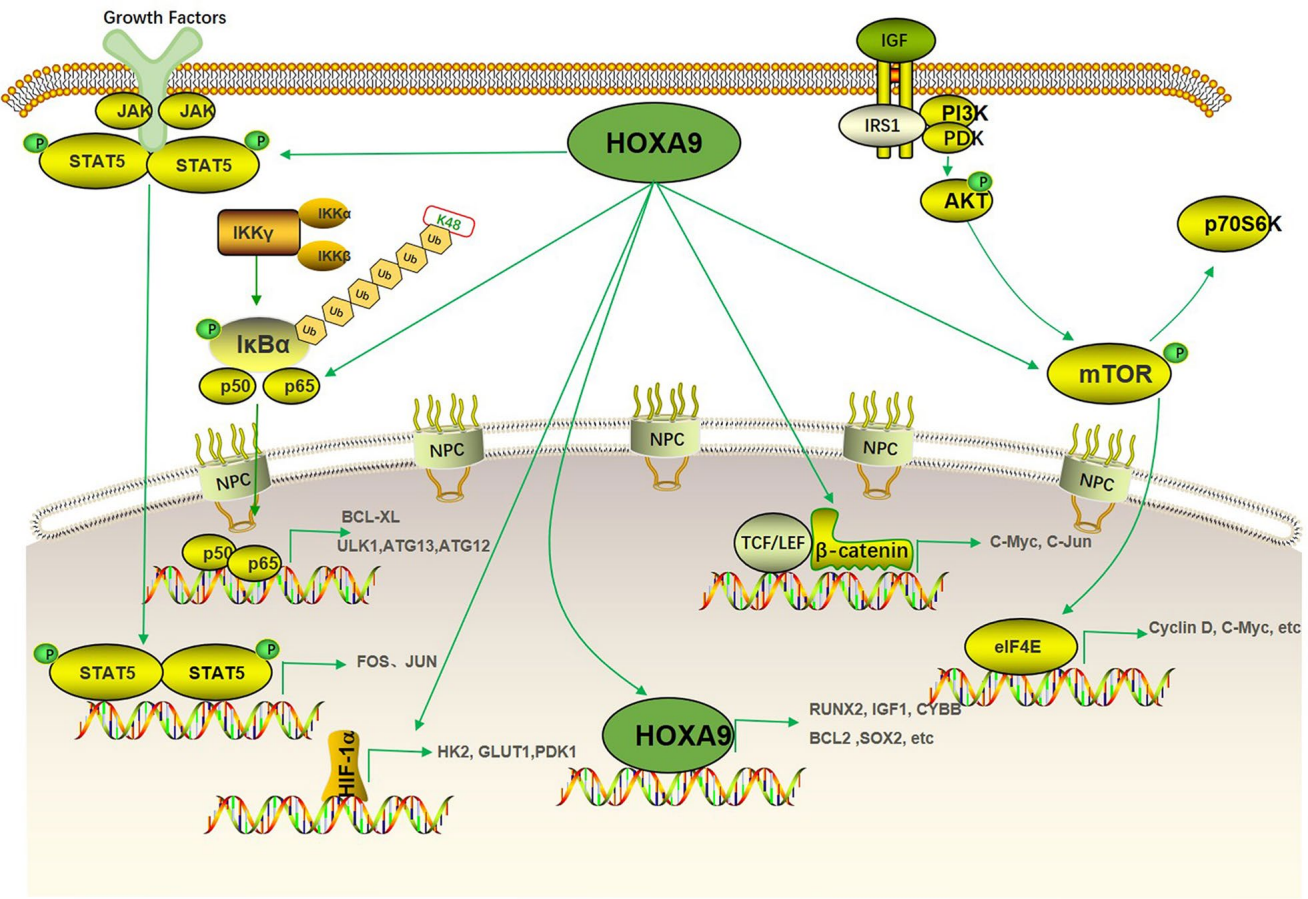
Schematic diagram of HOXA9-mediated signaling pathways. HOXA9 as a transcription factor can regulate multiple genes involving in proliferation, apoptosis, differentiation and metastasis such as RUNX2, IGF1, CYBB, BCL-2, SOX2 via binding to their promoters. Abnormal expression of HOXA9 is associated with multiple signaling pathways. HOXA9 promotes apoptosis and represses autophagy through regulating genes RELA, BCL-XL, ULK1, ATG3, and ATG12 associated with NF-κB signaling pathway. HOXA9 regulates glycolysis through blocking HIF-1a binding to glucose metabolism associated genes such as GLUT1, PGK1, and PDK1. HOXA9 was also associated with JAK/STAT pathway through enhancing the transcription activity of STAT5, leading to the upregulation of its downstream target genes FOS, JUN. Deregulation of HOXA9 is often accompanied by alteration of Wnt/β-catenin signaling. HOXA9 as a direct target of miR-429 regulates Wnt/β-catenin signaling pathway through regulation of β-catenin, c-myc, c-jun expression. Abnormal expression of HOXA9 also leads to the alteration of PI3K/Akt signaling pathway. Overexpression of HOXA9 promotes the ability of proliferation, invasion, and migration through upregulating the expression of p-PI3K, p-Akt, Cyclin D, C-Myc, MMP2, and MMP9

The classical function of HOXA9 is that it mainly regulates genes expression as a transcription factor (Fig. 1). Take an example, Jin et al. discovered that HOXA9 can bind at the promoter of RUNX2, and enhance calcification, migration, and invasion in papillary thyroid carcinoma [17]. In addition, IGF1 is confirmed as a direct target of HOXA9 through binding at the 90 kb upstream of the transcription start site of IGF1 tested by ChIP experiment [18]. What’s more, HOXA9 regulates multiple genes expression through binding to their promoters such as CYBB [19], CDX4 [20], BCL2, SOX4 [21], E-selectin [22], LRRFIP1 [23], Flt3 [24], WNT6 [25], Pim1 [26], BRCA1 [27]. While the cross-talk between HOXA9 and its target is worthy to be further investigated. Although HOXA9 working as a transcription factor engages in various biological events in organisms, HOXA9 might have a non-transcriptional function which is also important owing to its role in human diseases. HOXA9 as an E3 ubiquitin ligase leads to the ubiquitination degradation of Geminin which is a DNA replication inhibitor [28]. While the conflicting reports found that the binding of HOXA9-Geminin sequesters HOXA9 leads to the inhibition of its transcriptional activity [29]. More explanation about the function of HOXA9 in solid tumors is warranted.

## Deregulation of HOXA9 in solid tumors

Aberrant expression of HOXA9 is not only presented in AML, but also observed in various solid tumors, making it an interesting and potential target in tumor therapy. For example, the activation of HOXA9 is regarded as a novel, independent, and negatively prognostic marker in malignant glioblastoma (GBM) patients [30]. In colon cancer (CRC), HOXA9 expression is elevated in CRC tissues compared to the normal epithelium, HOXA9 might drive CRC development and growth through regulating stem cell (SC) function [31]. In addition, the function of HOXA9 in Nasopharyngeal carcinoma (NPC) is also explored. Researchers found that HOXA9 expression was significantly higher in NPC than in control tissues, and correlated positively with the clinical stage and T stage of NPC. Besides, the prognosis was much poorer in NPC patients which showing higher expression of HOXA9 [32]. While the specific mechanism of HOXA9 in NPC needs further study.

What’s should be noted that HOXA9 not only acts a role of oncogene but also a tumor suppressor gene. Zhou et al. reported that HOXA9 is identified to be downregulated in cutaneous squamous cell carcinoma (cSCC), and HOXA9 acting as a tumor suppressor impedes the binding of HIF-1α, thus repressing it downstream gene expression [33]. HOXA9 was found to be downregulated in breast cancers, and reduced HOXA9 expression was linked to the malignant phenotype of breast tumor. Inhibition of HOXA9 resulted in the epithelial cell growth, survival, and disturbed tissue morphogenesis, while recovering HOXA9 expression had the opposite role. Mechanistically, HOXA9 inhibited breast tumor malignant behavior through modulating BRCA1 expression [27]. This prompts us that HOXA9 expression in tumors might have heterogeneity, and research on the role of HOXA9 in various tumors depends on the tumor itself.

## Multiple signaling pathways influenced by HOXA9

Aberrant activation of multiple signaling pathways associated with HOXA9 presented in various solid tumors such as the NF-κB, HIF-1α, JAK/STAT, Wnt/β-catenin, and PI3K/AKT/mTOR signaling pathway leads to uncontrolled cell proliferation, invasion, and apoptosis (Fig. 1). Mounting evidence has shown that dysregulated HOXA9 expression linked to the following signaling pathways, focused on the signaling pathways regulated by HOXA9 is benefit for comprehending the mechanisms of HOXA9 on solid tumors.

## NF-κB signaling pathway

NF-κB signaling pathway is a classical pathway which plays vital role in the organ. It is well-known that NF-κB is activated in responding to a series of stimuli such as inflammation, proliferation, and apoptosis [34]. The activation of the canonical NF-κB is mainly depends on the phosphorylation of the IKKs complex. The phosphorylated IKKβ then promotes the phosphorylation of IκB which results in the K48-linked ubiquitination degradation of IκBs, leading to the translocation of NF-κB dimers from cytoplasmic to the nucleus, and finally driving the transcription of downstream target genes [35]. Deregulation of NF-κB signaling pathway has been reported to engage in multiple tumors such as lung cancer [36], gastric cancer [37], and hepatocellular carcinoma [38]. Interestingly, deregulation of HOXA9 was found to be associated with NF-κB signaling pathway. HOXA9 promotes apoptosis and represses autophagy through transcriptionally regulating the p65 subunit of NF-κB RELA in cSCC [39]. The responsive region of NF-κB is described to locate in the first 400 bp of the promoter of HOXA9 [40]. In a model of non-small cell lung cancer (NSCLC), HOXA9 was downregulated in lung tumor tissues when compared with matched non-tumor tissues, and HOXA9 overexpression inhibited cell migration and invasion which is also associated with the regulation of NF-κB activity [41]. While there are inconsistent in evidence that HOXA9 overexpression promoted the activation of NF-κB signaling reported by Jiahui Zeng et al. [42]. A possible explanation for this contradiction is that there might exist cell heterogeneity. In general, the precise mechanisms of how HOXA9 activates the NF-κB signaling pathway warrants further deep study.

## HIF-1α signaling pathway

Hypoxia-inducible factor (HIF-1) is a transcriptional activator depending on the availability of oxygen. The HIF family is made up of two subunits including α and β subunit. The α subunit contains HIF-1α, HIF-2α, and HIF-3α, while the β subunit has just one protein (HIF-1β) [43]. The expression level of the α subunit is significantly higher in hypoxia condition than in normoxia condition. Under normoxia condition, the HIF-1α subunit is increasingly degraded through ubiquitin–proteasome pathway mediated by the von Hippel-Lindau tumor suppressor gene (pVHL). HIF-1α plays a vital role in physiological homeostasis. Take an example, research reported that HIF-1 reprogram glucose metabolism through regulating the transcription activity of glucose-associated genes such as GLUT1, PGK1, and PDK1 [44]. Zhou et al. reported that loss of HOXA9 inhibits glycolysis in cSCC by hindering the binding of HIF-1α to its downstream targets such as HK2, GLUT1, and PDK1. In detail, HOXA9 interacted with CRIP2 at the promoter of glycolytic genes impeding the binding between HIF-1α and glucose metabolism-associated genes [33]. HOXA9 as an oncogene upregulated in Head and neck squamous cell carcinoma (HNSCC) is also reported to be related with HIF-1a signaling pathway. HOXA9 knockdown suppressed cell malignant events in CAL-27 and KB cells, and HOXA9 as a downstream target was transcriptionally regulated by HIF-1a [45]. In addition, Xia et al. found that miR-652 promoted HIF-1alpha signaling through inhibition of HOXA9 expression in uveal melanoma [46]. The above evidence showed that HOXA9 was associated with HIF-1a signaling, targeting this HOXA9- HIF-1a axis and its downstream targets might be valuable in tumor therapy.

## JAK/STAT signaling pathway

Janus kinase-signal transducer and activator of transcription (JAK-STAT) signaling has been identified as a key evolutionarily conserved cellular mechanism in transducing extracellular signals into intracellular transcriptional programs regulating cell growth and differentiation [47]. Aberrant activation of JAK/STAT signaling has been discovered to contribute to tumorigenesis. Zhu et al. confirmed that zip inhibition promoted JAK/STAT3 activation in tamoxifen-resistant breast cancer [48]. Wei Huang also reported that the abnormal activation of the JAK/STAT signaling pathway in bladder cancer induced by IGF2BP3, and the JAK/STAT signaling inhibitors markedly hindered the activity of IGF2BP3 [49]. What we are interested in is whether HOXA9 could regulate JAK/STAT pathway. There was a piece of strong evidence showed that HOXA9 was associated with JAK/STAT pathway. In detail, HOXA9 cooperates with activated JAK3/STAT5 signaling drives the progression of T-cell acute lymphoblastic leukemia through integrating RNA sequencing, chromatin immunoprecipitation sequencing, and assay for Transposase-Accessible Chromatin using sequencing (ATAC-seq) [50]. Similar research also confirmed that HOXA9 cooperated with STAT5 locates in the same genomic loci resulting in an increased JAK/STAT signaling [51]. Related evidence also showed that HOXA9 was induced in aged mice, which in turn activates several developmental pathways including Wnt, TGFβ, and JAK/STAT signaling pathway [52]. While the exact mechanism on HOXA9 associated with JAK/STAT signaling pathway in solid tumors is further needed.

## Wnt/β-catenin signaling pathway

It was found that the canonical Wnt/β-catenin signaling axis was linked to tumorigenesis [53]. Deregulation of HOXA9 is often accompanied by alteration of Wnt/β-catenin signaling. Based on the published literature, we found an interesting point that HOXA9 is the target of a series of miRNAs which will be introduced in the later chapter associated with Wnt/β-catenin signaling axis. For instance, HOXA9 is a direct target of miRNA-429 which was verified by luciferase reporter assay, and miR-429 inhibited OS progression through targeting HOXA9 via Wnt/β-catenin signaling pathway [54]. Xu et al. also reported that HOXA9 overexpressed damnified the antitumor effect of miR-638 in breast cancer via Wnt/β-catenin signaling pathway [55]. Thus, it can be seen that there exists a closely relationship between HOXA9 with Wnt/β-catenin axis. However, further investigation into the relationship between the Wnt/β-catenin pathway and HOXA9 will be benefit for tumor treatment.

## PI3K/AKT/mTOR signaling pathway

The abnormal activation of PI3K/AKT/mTOR signaling axis was often observed in the biological processes such as cell metabolism, proliferation, and apoptosis [56]. PI3K activation is stimulated by various growth factor receptor tyrosine kinases including ERBB receptors, FGFR, and GPCR, etc. Activated PI3K phosphorylates PIP2 and converts PIP2 to PIP3 at cell membrane. PIP3 acting as a docking for AKT recruits PDK1 and AKT via pleckstrin homology (PH) domain. PDK1 interacts with AKT and phosphorylates AKT at Thr308 resulting in the activation of AKT, and the activation of AKT promotes cell growth through phosphorylating proteins in the cytoplasmic. Among these proteins, the central downstream effector is the serine/threonine kinase mTOR which is sensitive to nutrient conditions and mediates cell catabolic and anabolic processes in order to support the balance of organism. Once AKT is activated, it inhibits tuberous sclerosis complex 1 and 2 (TSC1/2) through phosphorylation, leading to the activation of the complex mTOR/Raptor (TORC1). TORC1 activation promotes the phosphorylation of ribosomal protein S6 kinase (S6K1) and eIF4E binding protein 1 (4E-BP1), therefore facilitating messenger RNA translation and protein synthesis [57].

Abnormal expression of HOXA9 also leads to the alteration of PI3K/Akt signaling pathway. It was reported that overexpression of HOXA9 promotes the ability of proliferation, invasion, and migration in osteosarcoma cells through upregulating the protein expression of p-PI3K, p-Akt, MMP2, and MMP9 [58]. The research of Costa et al. also showed that the PI3K/AKT/mTOR signaling pathway is a critical regulatory mechanism of HOXA9 gene expression in GBM, and PI3K inhibitor LY294002 treated in A172 cells leads to a reduction of HOXA9 expression, which is associated with reversible epigenetic histone modifications [30]. These results confirmed the significant role regulated by the PI3K/AKT signaling axis in the HOXA9-mediated tumor progression.

## HOXA9 binding partners

The binding partners of HOXA9 were also summarized in this review (Table 1), which might be helpful in understanding the mechanism of HOXA9 in various tumors and presenting a potential means to target HOXA9 based on protein–protein interaction.

**Table 1:** Summary of HOXA9 binding partners and its mechanisms

Binding partners	Mechanisms	PMID	References
PBX3	PBX3 showed a synergistic effect with HOXA9 in inducing leukemia, and small peptide HXR9 disrupted the PBX3/HOXA9 interaction leading to the inhibition of cell proliferation and promotes apoptosis in AML cells	23264595	[59]
MEIS1	HOXA9 physically interacted with MEIS1 which is also a cofactor of HOXA9, SCUBE1 is confirmed as a novel target of HOXA9/MEIS1invoving in activation of the FLT3-LYN signaling axis	36005562	[66]
JMJD1C	JMJD1C aggravates AML malignancy through demethylase-independent upregulation of glycolytic and oxidative associated genes in HOXA9-dependent AML, JMJD1C coexpressed and interacted with HOXA9	30622285	[68, 69]
PRMT5	PRMT5 is recruited to the promoter of E-selectin, HOXA9 is transiently bind to their cognate recognition sequence, and induces the symmetric dimethylation of HOXA9 at Arg140, which is crucial for the induction of E-selectin	22269951	[71]
Smad4	HOXA9 interacted with Smad4 in the cytoplasmic and protected the transformation of normal HSPCs induced by HOXA9, and ruined their interaction with truncated Smad4 leads to the increasing of its target genes such as p15, p21, p27, activates the TGF-β signaling pathway and induces apoptosis in leukemic stem cells(LSCs)	21471525	[72]
G9a	G9a facilitated the gene expression of HOXA9-dependent in mouse AML cells through interacting with HOXA9 and recruiting to the sites of HOXA9-dependent genes, and G9a inhibition inhibited AML cell proliferation and induced differentiation	24532712	[73]
EIF4E	HOXA9 promotes the export of cyclin D1 and ornithine decarboxylase (ODC) mRNAs in the nucleus and increases the translation efficiency of ODC mRNA in the cytoplasm through directly interacting with EIF4E and competing with PRH from EIF4E	15657436	[75]
C/EBPα, Creb1, Stat5	Immunoprecipitations assay confirmed that other proteins such as C/EBPα, Creb1, and Stat5 also interacted with HOXA9 to increase acetylation and coactivator recruitment, while further deeper mechanisms about the role of these binding partners in human diseases mediated by HOXA9 are required	22072553	[76]

PBX family proteins are described to raise the DNA-binding/transcriptional activity of HOX family. Research reported that PBX3 is co-expressed with HOXA9 in AML, and presenting a therapeutic potential in targeting the interaction between PBX3 and HOXA9. A small and unique peptide HXR9 was generated to destroy the interaction between HOX and PBX protein, which selectively damages leukemic cells [59]. In addition to treating AML, researches on HXR9 used in solid tumor such as melanoma, ovarian cancer [60], breast cancer [61] were also emerged. Take an example about this interaction abrogated by HXR9, the small HXR9 peptide hinders the binding ability of HOXA9 to PBX, resulting in an inhibition of meningioma growth [62]. However, the small peptide HXR9 is not constrained to disrupt the HOXA9/PBX3 interaction, the HOX/PBX proteins such as HOXB7/PBX2 interaction which is positively regulated by microRNA-221 and -222 was also disrupted by HXR9 treated in melanoma [63]. At present, no clinical trial about HXR9 in human treatment was reported. More preclinical and clinical evidence on HXR9 are required in the near future. It was reported that HOXA9 and MEIS1 co-expressed in murine marrow causes the rapidly myeloid leukemia progression [64]. HJ Lawrence also provided direct evidence that HOXA9 interacted with MEIS1 [65]. Xinyue Zhou and Rui Lu discovered that SCUBE1 involving in the initiation and maintenance of MLL-rearranged (MLL-r) acute myeloid leukemia is a novel transcriptional target of HOXA9 and MEIS1 [66], understanding the role of SCUBE1 is contributed to clarify the mechanism of HOXA9/MEIS1in human diseases. It should be noted that PBX3 and MEIS1 cooperated with HOXA9, Garcia-Cuellar et al. showed that destroyed the dimerization of MEIS1/PBX3 can abrogate the ability of proliferation in primary cells transformed by HOXA9 [67]. JMJD1C is a member of the lysine demethylase 3 (KDM3) family which contains jumonji domain. Zhu et al. discovered that HOXA9 drove gene expression in leukemia stem cells depending on JMJD1C and directly interacted with JMJD1C [68]. In addition, JMJD1C-mediated metabolism dysregulation contributes to HOXA9-dependent AML [69]. PRMT5 is a member of the PRMT family which govern multiple cellular processes through catalyzing protein arginine methylation. PRMT5 is a constituent of various protein complexes and participates in multiple cellular biological processes including RNA transport, tumor growth, and chromatin remodeling [70]. Bandyopadhyay et al. discovered that PRMT5 is one of the HOXA9 binding partners detected by mass spectrometry (MS). Mechanically, PRMT5 is recruited to the promoter of E-selectin, HOXA9 is then transiently bind to their cognate recognition sequence, and induces the symmetric dimethylation of HOXA9 at Arg140, which is crucial for the induction of E-selectin [71]. Smad4 as a tumor suppressor was a binding partner of HOXA9, Quéré et al. found that HOXA9 interacted with Smad4 in the cytoplasmic and protected the transformation of normal HSPCs induced by HOXA9, and ruined their interaction with truncated Smad4 leads to the increasing of its target genes such as p15, p21, p27, activates the TGF-β signaling pathway and induces apoptosis in leukemic stem cells(LSCs) [72]. In addition to TGF-β signaling pathway, whether other signaling pathways were also affected by the truncated Smad4 is worthy to be explored. Following study about the specific position of Smad4 which is responsible for HOXA9 binding is also required. Lysine methyltransferase G9a, an attractive target in treating various tumors, is responsible for regulating gene transcription through catalyzing methylation of histone H3 lysine 9. Lehnertz et al. confirmed that G9a facilitated the gene expression of HOXA9-dependent in mouse AML cells through interacting with HOXA9 and recruiting to the sites of HOXA9-dependent genes, and G9a inhibition inhibited AML cell proliferation and induced differentiation [73], suggesting that G9a is a potential target in AML and solid tumors. In fact, it has attracted a lot of attention on the development of G9A inhibitors in human diseases. Take an example of G9A inhibitor, BIX01294 reduced tumor growth and metastasis in Ewing sarcoma through upregulating NEU1 [74]. However, no available inhibitors of G9a were successfully passed through the early stages of clinical testing. More G9A inhibitors will be introduced in the following section of pharmacological targeting HOXA9. EIF4E acting in the rate-limiting step of translation initiation alters gene expression on multiple levels, HOXA9 promotes the export of cyclin D1 and ornithine decarboxylase (ODC) mRNAs in the nucleus and increases the translation efficiency of ODC mRNA in the cytoplasm through directly interacting with EIF4E and competing with PRH from EIF4E [75]. However, the research is lack of animal experiments, and more evidence about the mechanism of HOXA9 and EIF4E were necessary to be achieved. In addition to the above proteins binding to HOXA9, Immunoprecipitation assay confirmed that other proteins such as C/EBPα, Creb1, and Stat5 also interacted with HOXA9 in order to increase acetylation and coactivator recruitment [76], while further deeper mechanisms about the role of these binding partners in human diseases mediated by HOXA9 are required. Above those proteins that were reported to bind HOXA9, it is worth thinking about what we can do according to these HOXA9 binding partners. Understanding the interaction and function role of HOXA9 and its binding partners is benefit for developing potential drug targets in cancer therapy.

## Post-transcriptional regulation of HOXA9

In addition to be regulated at transcriptional levels, many post-transcriptional events including microRNAs, long non-coding RNAs, and epigenetic modification could also determine HOXA9 protein level and function. These post-transcriptional events show alternative aspects of HOXA9 regulation and might offer novel therapeutic opportunities in cancer treatment.

## HOXA9 regulated by microRNAs

MicroRNAs (miRNAs) are a type of small and short RNAs which have critical roles in regulating the post-transcriptional of target genes. miRNAs are associated with the etiology mechanism of multiple diseases including asthma, allergic rhinitis, and tumor [77]. Retrieval of the published literature on HOXA9, we found that many documents involved the role of HOXA9 regulated by multiple microRNAs in various tumors (Table 2). In addition, we observed that study about HOXA9 as a target of these miRNAs in osteosarcoma is more than in other solid tumors, prompting that HOXA9 might implement a crucial role in osteosarcoma regulated by miRNAs. Research reported that HOXA9 was identified as a downstream target of miR-1294 in osteosarcoma [78]. Furthermore, HOXA9 as a direct target of miR-429 [54], miR-873 [79], miR-652 [58], miR-182 [80], miR-641 [81], and miR-139-5p [82] were also verified by luciferase reporter assay in osteosarcoma. In glioma, miR-638, miR-647 as tumor suppressors regulate cellular malignancy of gliomas by targeting HOXA9 [83, 84]. Among these miRNAs, miR-182-5p [85], miR-196p [41], and miR-19b-3p [86] exert oncogenic role in NSCLC through regulating HOXA9. Furthermore, miR-633 in colorectal adenocarcinoma [87], miR-381-3p in human anaplastic thyroid carcinoma [88], microRNA-638 in breast cancer [55], miR-652 in Uveal Melanoma [46], miR-133b in colorectal cancer [89], and miR-139-5p in oral squamous carcinoma [90] were found to regulate the malignant behavior through targeting HOXA9. While the role of those miRNAs regulated by HOXA9 in other tumors is unclear and intensive studies about the detailing mechanism of HOXA9 regulated by miRNAs such as the downstream signaling pathways and upstream modulators in solid tumors is needed in the future. Therefore, the above research on the regulation of HOXA9 by miRNAs leads us to think that targeting miRNA/HOXA9 axis might have therapeutic potential in various solid tumors, while further mechanism investigation on the role of HOXA9 regulated by miRNAs is required.

**Table 2:** Summary of HOXA9 as targets of miRNAs in tumorigenesis and related function

miRNAs	Tumor type	Target	Functions	PMID	References
miR-1294	Osteosarcoma	HOXA9	miR-1294 functions as a tumor suppressor in OS progression by targeting HOXA9	30575897	[78]
miR-429	Osteosarcoma	HOXA9	miR-429 suppressed OS progression by targeting HOXA9 through Wnt/β-catenin pathway	32782562	[54]
miR-873	Osteosarcoma	HOXA9	miR-873 suppresses the development of OS by directly targeting HOXA9 and inhibiting the Wnt/β-catenin pathway	30816476	[79]
miR-652	Osteosarcoma	HOXA9	miR-652 may negatively regulate HOXA9 expression and inhibit the proliferation, migration, and invasion abilities of osteosarcoma cells through the PI3K/Akt signaling pathway	35111226	[58]
miR-641	Osteosarcoma	HOXA9	miR-641 was found to target the 3' untranslated region of homeobox protein Hox-A9 (HOXA9) and suppressed the expression of HOXA9	30838699	[81]
miR-182	Osteosarcoma	HOXA9	miR-182 downregulates Wnt/β-catenin signaling, inhibits cell proliferation, and promotes apoptosis in osteosarcoma cells by suppressing HOXA9 expression	29254169	[80]
miR-139-5p	Osteosarcoma	HOXA9	miR-139-5p depressed the invasion, proliferation, and EMT of osteosarcoma cells via targeting HOXA9	35800618	[80]
miR-638	Glioma	HOXA9	miR-638 as a tumor suppressor regulates cellular malignancy of gliomas through targeting HOXA9	30536324	[83]
miR-647	Glioma	HOXA9	HOXA9 was validated a direct target of tumor suppressor miR-647	31881106	[84]
miR-182-5p	Non-small cell lung cancer	HOXA9	MiR-182-5p may exert oncogenic influence on NSCLC through regulating target genes such as HOXA9	31906958	[85]
miR-196p	Non-small cell lung cancer	HOXA9	HOXA9 expression is inversely correlated with miR-196b levels in clinical NSCLC samples as compared to that in corresponding control samples	26586336	[41]
miR-19b-3p	Non-small cell lung cancer	HOXA9	High expression of miR-19b-3p was found to be capable of predicting poor clinical prognosis in NSCLC patients	35274735	[86]
miR-633	Colorectal adenocarcinoma	HOXA9	PCED1B-AS1 could negatively regulate the expression of HOXA9 by sponging miR-633	35176937	[87]
miR-381-3p	Human anaplastic thyroid carcinoma	HOXA9	miR-381-3p may function as a tumour suppressor to impede proliferation, migration and invasion and induce apoptosis of DOX-resistant SW1736 and CAL62 cells by inhibiting HOXA9 protein expression	34719830	[88]
miR-638	Breast cancer	HOXA9	miR-638 inhibits breast cancer progression through binding to HOXA9	34416888	[55]
miR-652	Uveal Melanoma	HOXA9	miR-652 as an oncogene promoted HIF-1alpha signaling via repression of HOXA9 in uveal melanoma cells	31740654	[46]
miR-133b	Colorectal cancer	HOXA9	miR-133b targeted the HOXA9/ZEB1 pathway to promote tumor metastasis in CRC cells	28969042	[89]
miR-139-5p	Oral squamous carcinoma	HOXA9	miR-139-5p could directly inhibit HOXA9, which might be a potential mechanism in inhibiting the proliferation, invasiveness and migration of OSCC cells	28780773	[90]

## HOXA9 regulated by long non-coding RNAs

Long non-coding RNA(lncRNAs) are transcripts of over 200 nucleotides which don’t code proteins [91]. Abnormal expression of lncRNA might play direct impact on cell signaling cascades in tumor progression through interacting with other RNA species or proteins [92]. The emerging roles of these lncRNAs in the field of tumor biology is can’t be ignored. Among the topic we focused on in this review, it was found that HOXA9 is also regulated by a series of long non-coding RNAs (Table 3). Liu et al. discovered that lncRNA PCED1B-AS1 enhances colorectal adenocarcinoma progression through negatively controlling the expression of HOXA9 by sponging miR-633 [87]. Ana Xavier-Magalhães explored the upstream regulatory mechanism of lncRNA HOTAIR in gliomas. Their results shown that both lncRNA HOTAIR and HOXA9 were co-expressed in gliomas with high-grade, and further HOXA9 binds directly to the promoter of HOTAIR [93]. Not only does HOTTIP regulates HOXA9, but also regulates other HOX genes such as HOXA13, HOXA11, and HOXA1, leading us to think that lncRNA HOTTIP plays an essential role in tumors through HOX gene family [94–96]. TWIST1 functioning as a transcription factor co-expressed with HOXA9 was activated in prostate tumors, research further showed that TWIST1, WDR5 and the lncRNA HOTTIP formed a complex and regulated the chromatin in the promoter of HOXA9 [96]. Furthermore, LINC01140 and LncRNA DLX6-AS1 were reported to regulate the malignant behaviors including proliferation, invasion, and metastasis in Osteosarcoma through targeting HOXA9 [81, 82]. Triple-negative breast cancer (TNBC) is a highly invasive subtype of breast cancer, HOXA9 was reported to be regulated by LncRNA MIR503HG in TNBC through miR-224-5p [97]. What’s more, research about LncRNA ST8SIA6-AS1in Pituitary adenoma [98] and LncRNA KCNQ1OT1 in Laryngeal squamous cell carcinoma [99] were also associated with the regulation of HOXA9. In addition to being regulated in various tumors, LncRNA PEG10 and LncRNA UCA1 were reported to regulated HOXA9 in Cardiac hypertrophy [100, 101]. What’s should be noted that the mechanism on HOXA9 regulated by those LncRNAs is not deep enough, more investigation about lncRNA/HOXA9 axis in tumors is conducted in the future.

**Table 3:** Summary of HOXA9 associated with LncRNAs in human diseases and related function

LincRNAs	Tumor type	Target	Functions	PMID	References
PEG10	Cardiac hypertrophy	HOXA9	PEG10 aggravates cardiac hypertrophy by positively regulating HOXA9	31389601	[100]
HOTTIP	Prostate Cancer,Pancreatic cancer	HOXA9	HOTTIP mediated HOXA9 to enhance the Wnt/β-catenin pathway by binding to WDR5 in PCSCs	28484075; 28947139	[94–96]
MIR503HG	Triple-negative breast cancer	HOXA9	MIR503HG inhibited cell proliferation and promoted the apoptosis of TNBC cells via the miR-224-5p/HOXA9 axis, which may function as a novel target for the treatment of TNBC	33869743	[97]
ST8SIA6-AS1	Pituitary adenoma	HOXA9	ST8SIA6-AS1 targets miR-5195-3p to regulate the expression of HOXA9 and promote the EMT of pituitary adenomas	35783150	[96]
UCA1	Cardiac hypertrophy	HOXA9	UCA1 promoted the progression of cardiac hypertrophy through competitively binding with miR-184 to enhance the expression of HOXA9	29616999	[101]
DLX6-AS1	Osteosarcoma	HOXA9	DLX6-AS1 functions as a ceRNA by targeting miR-641/HOXA9 signal pathway to suppress OS cell proliferation and metastasis	30838699	[81]
LINC01140	Osteosarcoma	HOXA9	LINC01140 downregulation inhibits the invasion, proliferation, and EMT in osteosarcoma cells through targeting the miR-139-5p/HOXA9 axis	35800618	[80]
PCED1B-AS1	Colorectal adenocarcinoma	HOXA9	PCED1B-AS1 could negatively regulate the expression of HOXA9 by sponging miR-633	35176937	[87]
HOTAIR	Glioma	HOXA9	HOXA9 as a novel direct regulator of HOTAIR, and establishes HOTAIR as an independent prognostic marker, providing new therapeutic opportunities to treat this highly aggressive cancer	29644006	[93]
KCNQ1OT1	Laryngeal squamous cell carcinoma	HOXA9	ALKBH5-mediated m6A modification of KCNQ1OT1 triggers the development of LSCC via upregulation of HOXA9	34850551	[99]

## HOXA9 Methylation

DNA methylation is a kind of epigenetic mechanisms which govern multiple biological processes in eukaryotes such as proliferation, apoptosis, and differentiation. It is measurable with dinucleotides located in the nearly eighty percent of the CpG island in human genome. Aberrant DNA methylation is often associated with malignant events in tumors depending on DNA hypermethylation or hypomethylation [102]. HOXA9 methylation has been observed in various tumors. HOXA9 methylation occurred in hepatocellular carcinoma tissues which might be a useful biomarker in detection of hepatocellular carcinoma [103]. HOXA9 was remarkable more hypermethylated in patients with lung cancer compared with benign lung diseases and the healthy group [104]. HOXA9 promoter methylation was found in ovarian cancer revealed that the potential of HOXA9 methylation is a good diagnostic biomarker used in early screening of ovarian cancer [105]. What’s more, A meta-analysis conducted by Cai et al. found that HOXA9 methylation has a significantly estimable biomarker for predicting poor prognosis and is a potential target for therapy in solid malignant carcinoma [106]. A large amount of research on HOXA9 methylation in various tumors showed that a useful biomarker of HOXA9 methylation in the early detection of solid tumors, while a deeper mechanism on that is still needed.

## Pharmacological targeting HOXA9

The important role of HOXA9 in AML and solid tumors make it a potential target in cancer therapy. Indeed, Mélanie Lambert et al. summarized the detailed information about small inhibitors targeting HOXA9 directly and indirectly [107]. In our review, we also introduced the mechanisms of part of HOXA9 inhibitors which were listed in Table 4.

**Table 4:** List of developed inhibitors for pharmacological targeting of HOXA9 presented in this review

Targets	Inhibitors	Stage of development	PMID	References
DOT1L inhibitors	EPZ004777	Preclinical Stage	21741596	[109]
	SGC0946	Preclinical Stage	23250418	[110]
	EPZ-5676	Phase I(NCT02141828)	31790636	[113]
	SYC-522	Preclinical Stage	24858818	[115]
LSD1 inhibitors	TCP	Phase I(NCT02273102)	35914778; 32561840	[118, 119]
	ORY-1001	Phase I(NCT05546580)	35914778; 29502954	[118, 120]
	GSK-2879552	Phase I/II(NCT02929498)	35914778; 29972300	[118, 121]
	INCB059872	Phase I(NCT03132324)	35914778	[118]
	IMG-7289	Phase II(NCT04254978)	35914778	[118]
	ORY-2001	Phase II(NCT03867253)	35914778	[118]
	SP2509	Preclinical Stage	24699304	[122]
	NCD38	Preclinical Stage	28210006	[123]
	JL1037	Preclinical Stage	28404874	[124]
	FY-56	Preclinical Stage	35051709	[125]
	Higenamine	Phase I(NCT01451229)	33618250	[126]
	MS142	Preclinical Stage	30679800	[127]
WDR5-MLL inhibitors	MM-101	Preclinical Stage	23210835	[129]
	MM-102	Preclinical Stage	23210835	[129]
	MM-103	Preclinical Stage	23210835	[129]
	MM-589	Preclinical Stage	28603984	[130]
	MM-401	Preclinical Stage	28603984	[130]
	DDO-2084	Preclinical Stage	27598236	[131]
	OICR-9429	Preclinical Stage	34154613	[132]
G9a inhibitors	DCG066	Preclinical Stage	27393948	[134]
	BIX-01294	Preclinical Stage	34876693	[135]
	UNC0638	Preclinical Stage	29207160	[136]
	UNC0642	Preclinical Stage	30765842	[137]
	A-366	Preclinical Stage	34830365	[139]
	CM-272	Preclinical Stage	28548080	[140]
Menin-MLL inhibitors	SNDX-5613	Phase I/II(NCT05406817)	35017466	[144]
	KO-539	Phase I/II(NCT04067336)	36151141	[145]
	JNJ-75276617	Phase I(NCT04811560)	–	–
	BMF-219	Phase I(NCT05153330)	–	–
	M-1121	Preclinical Stage	34196551	[146]
BET inhibitors	JQ-1	Preclinical Stage	35745584	[148]
	I-BET151	Preclinical Stage	35745584	[148]
	AZD5153	Phase I/II(NCT05253131)	35399501	[151]
	BMS-986158	Phase I(NCT03936465)	36077617	[152]
	BI894999	Phase I( NCT02516553)	35444289	[153]
	GS-5829	Phase I/II(NCT02983604)	35816286	[154]
	GSK525762	Phase II(NCT01943851)	35681742	[155]
	ABBV-075	Phase I(NCT02391480)	36063752	[156]
	CPI-0610	Phase I(NCT02157636)	27890933	[157]
	INCB057643	Phase I(NCT04279847)	33396954	[158]
	OTX-015	Phase I( NCT01713582)	31632543	[159]
	PLX51107	Phase I/II(NCT04910152)	33504139	[160]
	INCB054329	Phase I/II(NCT02431260)	33148670	[161]
	FT-1101	Phase I( NCT02543879)	33574760	[162]
	CC-90010	Phase I( NCT04047303)	32240793	[163]
	ODM-207	Phase I/II(NCT03035591)	32989226	[164]
CDK4/6 inhibitors	PD 0332991	Phase I(NCT01701375)	25744718	[166]
	Abemaciclib	Phase II(NCT04169074)	36174113	[170]
	Ribociclib	Phase II(NCT05163106)	35972817	[171]
	Lerociclib	Phase II(NCT05085002)	32130619	[172]
	Trilaciclib	Phase IV(NCT05071703)	34887261	[173]
	Dinaciclib	Phase I(NCT03484520)	34972202	[174]
	SHR6390	Phase I( NCT05103826)	33845905	[175]

## DOT1L inhibitors

DOT1L is a histone methyltransferase which is linked with cell development. Selective drugs developed to restrain the proliferation of MLL-r leukemia cells through inhibiting DOT1L due to the ability of inducing the hyperexpression of HOXA9 and MEIS1. EPZ004777, a specific SAM-competitive inhibitor of DOT1L, inhibited MTDH-induced proliferation, invasion and expression of angiogenic markers through promoting NF-κB translocated to the promoter of HIF1α and increasing its transcription in TNBC cells [108]. Although a potential therapeutic value exhibited by EPZ004777, it should be noted that the effectiveness of EPZ004777 in vivo is limited due to the short half-life, and thus ended at the preclinical stage [109]. SGC0946, a brominated analogue of EPZ004777, inhibit DOT1L in vitro and selectively kill mixed lineage leukaemia cells [110]. A novel DOT1L inhibitor pinometostat(EPZ-5676) was better than EPZ004777 in inhibition potency and half-life was developed [111]. DOT1L inhibitor pinometostat(EPZ-5676) [112] explored in Phase I clinical trials used to treat pediatric and adult patients with *MLL*-driven leukemia presented promising outcomes(NCT02141828) [113]. EPZ-5676 also an inhibitor of DOT1L can actively destroy the proliferation ability of NPMc+ human leukemic cell through inhibiting the expression of HOXA9 and PBX3 [114]. SYC-522, a potent DOT1L inhibitor, was developed to inhibit the methylation at H3K79, decrease the expression of HOXA9 and MEISI and raise the sensitivity of MLL-rearranged leukemia cells presenting a potential means to treat MLL-rearranged leukemia [115]. Extensive investigation about this compound in the future is required due to the characteristic of the poor aqueous solubility and rapid metabolism. More DOT1L inhibitors are in the stage of preclinical. Although these DOT1L inhibitors were used in leukemia treatment, they might be potentially therapeutic based on their important role in solid tumors. More investigation about DOT1L inhibitors in solid tumors is required.

## LSD1 inhibitors

Lysine (K)-specific demethylase 1A (LSD1/KDM1A) is a lysine demethylase that was identified as a promising therapeutic target in tumor therapy through functioning on histones H3K4me1/2 and H3K9me1/2 [116]. LSD1-kd upregulated a series of critical hematopoietic genes including Gfi1b, HOXA9, and Meis1 in the HSC/progenitor compartment [117]. Over the last decades, large quantities of LSD1 inhibitors were developed. Six trans-2-phenylcyclopropylamine (TCP)-based LSD1 inhibitors including TCP, ORY-1001, GSK-2879552, INCB059872, IMG-7289, and ORY-2001 binding to the flavin adenine dinucleotide (FAD) with the catalytic cavity of LSD1 have already entered into clinical trials [118]. Tranylcypromine (TCP), one of the most potent LSD1 inhibitor has been widely studied. In a pilot trial of phase I/II, Tranylcypromine (TCP) combined with all-trans-retinoic acid (ATRA) induced the differentiation of AML blasts and produced clinical effectiveness in patients with relapsed/refractory (r/r) AML [119]. THP-1 MPAL cell treated ORY-1001(Iadademstat) down-regulates the expression of HOXA9, HOXA10 and HOXA11, induces differentiation, and promotes the expression of epigenetic markers including CD11b, S100A12, and LY96 in MV(4-11) mice [120]. GSK2879552, another potential LSD inhibitor, was reported to decrease the expression of HOXA9 expression and weaken cell viability in MOLM-13 MPAL cell line [121]. Other LSD1 inhibitors such as SP2509 [122], NCD38 [123], JL1037 [124] were at the preclinical stage. What’s more, FY-56, a promising LSD1 inhibitor, weakened the proliferation ability of leukemia cells, induced the accumulation of H3K4me1/2 and the activation of p53, and reduced HOXA9 and MEIS1 mRNA levels, exhibiting a therapeutic potential in AML [125]. Another two LSD1 inhibitors Higenamine [126], tranylcypromine derivatives MS142 also showed to inhibit LSD1 activity [127], while further investigation on the role of these LSD inhibitors in solid tumors is required.

## WDR5-MLL inhibitors

HOXA9 expression is promoted by complexes composed of mixed lineage leukemia 1 (MLL1), a histone H3 lysine 4 (H3K4) methylransferase, and WD-40 repeat protein 5 (WDR5) [128]. Therefore, ruining the MLL1/WDR5 protein–protein interaction is a helpful means to indirectly target HOXA9. Peptidomimetics MM-101, MM-102, and MM-103 with improved activities were developed, and the compound MM-102 showed selectively restrains cell growth in leukemia cells with MLL1 fusion proteins through decreasing HoxA9 and Meis-1 expression, which was two key genes in MLL1 fusion proteins in leukemogenesis [129]. Besides, Karatas et al. design and synthetize a series of peptidomimetics based on WDR5/ MLL1 interaction, they found that MM-589 inhibits the proliferation of leukemia cells with an IC50 value of 0.9 nM, and the inhibition ratio is better than the previously reported compound MM-401 [130]. Another research conducted by Dong Dong Li found that the promising compound 30 (DDO-2084) significantly suppressed proliferation and promoted apoptosis of MV4-11 cells via decreasing HOXA9 and MEIS-1 expression [131]. OICR-9429 blocking WDR5/MLL interaction through binding the core peptide-binding pocket of WDR5 induces cell cycle arrested in G1/S phase and promotes apoptosis and sensitivity to cisplatin in bladder cancer cells. What’s more, OICR-9429 blocks the activation of PD-L1 induced by IFN-γ, which leads to the inhibition of immune evasion [132]. It is worth exploring the application of OICR-9429 in immune therapy in the future.

## G9a inhibitors

G9a (KMT1C, EHMT2) as a lysine methyltransferase (KMT) dimethylates lysine 9 of histone H3 (H3K9me2). The upregulation of G9a increases methylation, which significantly leads to the downregulation of various tumor suppressor genes. G9a has become a promising target in tumor therapy [133]. Interestingly, G9a regulates HOXA9-dependent transcription in a mouse model of AML [73]. What’s more, Kondengaden et al. observed that a new G9a inhibitor DCG066 inhibits cell growth, blocks the cell cycle and induces apoptosis in leukemia cell lines identified through structure-based virtual screening [134], which means that targeting HOXA9 might be indirectly achieved by targeting G9a in various solid tumors. As mentioned earlier, the development of G9a inhibitors has been broadly researched. Several G9a inhibitors such as BIX-01294 [135], UNC0638 [136], and UNC0642 [137] blocking the H3 substrate binding site of G9a were designed based on the core of quinazoline. While BIX-01294 and its analogs presented a highly cytotoxicity in cells, UNC0638 showed a poorly pharmacokinetics in vivo [138]. G9a inhibitor A-366 was developed based on indole core, which promotes apoptosis in penumbra cells and reduces the volume of photothrombotic stroke-induced cerebral infarction [139]. CM-272 as a first-in-class dual inhibitor of G9a/DNMTs inhibits cell vitality, promotes apoptosis and prolongs the survival of AML and DLBCL mouse model, proving to be a promising drug in tumor therapy [140]. Further study showed that CM-272 combined with cisplatin promotes the regression of bladder tumors, and the anti-tumor effect is also remarkable in CM-272 combined with PD-L1 group [141]. Despite the success of preclinical study showed by G9a inhibitors, no clinical trials about G9a inhibitors were reported in human diseases. Therefore, more novel G9a inhibitors and deeper mechanisms for G9a in solid tumors are warranted.

## Menin-MLL inhibitors

Menin encoded by the MEN1 gene exacerbates the progression of leukemia induced by mixed lineage leukemia (MLL)-derived fusion proteins (MLL-FPs) [142]. Small inhibitors designed based on the interaction between Menin-MLL indirectly targeted HOXA9 like WDR5/MLL. In fact, disrupting Menin -MLL interaction is a novel therapeutic strategy in acute leukemia carrying MLL-rearrangements (MLL1-r). It is thought that small molecule Menin-MLL inhibitors inhibit MLL-FP-induced leukemia by disrupting the Menin-MLL interaction and inhibiting Menin/MLL induced HOXA9 gene [143]. A Menin-MLL inhibitor, SNDX-5613, was reported to inhibit HOXA9/MEISI transcription and induce the differentiation of AML. It’s now being entered in phase I/II study for the treatment of patients with relapsed/refractory AML harboring MLL1-r or NPM1c [144]. KO-539 (ziftomenib), a clinical drug candidate, inhibiting cell growth of MOLM13 and OCI-AML3 cells is being in phase I/II clinical study [145]. Menin-MLL inhibitor JNJ-75276617 was initiated a phase 1 study to treat Acute Leukemias, Acute Myeloid Leukemia and Acute Lymphoblastic Leukemia in 2021(NCT04811560). BMF-219 is also reported to initiate a phase 1 study in December 10, 2021 (NCT05153330). Another MENIN inhibitor M-1121 which interacts with MENIN at Cysteine 329 in the MLL binding pocket and inhibits acute leukemia cell growth through downregulating HOXA9 and MEIS1 gene expression [146]. These MENIN inhibitors indirectly targeted HOXA9 and might be potentially used in solid tumors.

## BET inhibitors

Bromodomain and extra-terminal (BET) proteins are reader proteins binding to the histone tails of acetylated lysine which plays a critical role in controlling gene expression [147]. BET inhibitors have been proved to be highly efficacy in a variety of tumors, and a number of BET inhibitors such as JQ-1 and I-BET151 were developed [148]. While some authors suggested that BRD4 inhibitors act dependently on HOX genes including HOXA9. Zha et al. demonstrated that BRD4 inhibitors JQ1 sensitized the curative effect of MOLM-13 and OCI-AML3 cells depending on HOXA9 gene expression [149]. Bardini et al. reported that another BET inhibitor I-BET151 blocked the growth of MLL-AF4+ leukemic cells through affecting the transcription level of BRD4, HOXA7/HOXA9, and RUNX1 [150]. At present, fourteen BET inhibitors including AZD5153 [151], BMS-986158 [152], BI894999 [153], GS-5829 [154], GSK525762 [155], ABBV-075 [156], CPI-0610 [157], INCB057643 [158], OTX-015 [159], PLX51107 [160], INCB054329 [161], FT-1101 [162], CC-90010 [163], and ODM-207 [164] have been entered in the clinical testing.

## CDK4/6 inhibitors

Cyclin-dependent kinase CDK4 and CDK6 are hyperactivated in various tumor. Developing small inhibitors based on CDK4/CDK6 is an alternative approach in tumor treatment [165]. PD 0332991 (palbociclib), is a selectively and potently inhibitor of CDK4 and CDK6. The research performed by Yang et al. showed that PD 0332991 induced apoptosis of AML cells through inhibiting HOXA9 expression and reducing its target PIM1expression [166]. The role of PD 0332991 in solid tumors such as breast cancer [167], lung cancer [168], and glioma [169] has been widely studied. Multiple clinical trials about palbociclib treated in various tumors such as breast cancer, ovarian cancer, Pancreatic Ductal Adenocarcinoma. Besides, other CDK4/6 inhibitors such as Abemaciclib [170], Ribociclib [171], Lerociclib [172], Trilaciclib [173], Dinaciclib [174], and SHR6390 [175] are also being adopted in clinical testing.

## Conclusions

This review described the structures of HOXA9 and its expression and diverse roles in solid tumors. The signaling pathways, post-transcription of modification, potential inhibitors, and binding partners of HOXA9 were presented. Although HOXA9 is a promising therapeutic target for AML and solid tumors, some issues need to be addressed. HOXA9 functioning as an oncogene or suppressor gene mainly depends on the heterogeneity of tumors. It is a challenge for us to investigate the mechanism of HOXA9 in tumors, especially in using HOXA9 inhibitors. In addition, the inhibitors of HOXA9 play a crucial role in tumor therapy through indirectly regulation of HOXA9 expression, no directly small molecular inhibitors targeting HOXA9 are developed at present. Therefore, the development of HOXA9 inhibitors is a topic needed to be focused on. What’s more, researches about HOXA9 in tumors were mainly focused on the miRNA, lncRNA, and methylation, while the mechanism is not deep enough. Further detailed study on the mechanistic roles of HOXA9 in tumors are required in the near future.

## Data Availability

Not applicable.

## Abbreviations

AML: Acute myeloid leukemia
miRNAs: MicroRNAs
ncRNAs: Non-coding RNAs
GBM: Glioblastoma
SC: Stem cell
CRC: Colon cancer
NPC: Nasopharyngeal carcinoma
cSCC: Cutaneous squamous cell carcinoma
NSCLC: Non-small cell lung cancer
HIF-1: Hypoxia-inducible factor
pVHL: Hippel-Lindau tumor suppressor gene
HNSCC: Head and neck squamous cell carcinoma
JAK-STAT: Janus kinase-signal transducer and activator of transcription
ATAC-seq: Transposase-accessible chromatin using sequencing
PH: Pleckstrin homology
TSC1/2: Tuberous sclerosis complex 1 and 2
S6K1: Ribosomal protein S6 kinase
4E-BP: EIF4E binding protein 1
KDM3: Lysine demethylase 3
MS: Mass spectrometry
LSCs: Leukemic stem cells
TNBC: Triple-negative breast cancer
WDR5: WD-40 repeat protein 5
H3K9me2: Lysine methyltransferase (KMT) di-methylates lysine 9 of histone H3
BET: Bromodomain and extra-terminal
